# Joint association of physical activity and sugar-sweetened beverages with obesity in young U.S. adults: A cross-sectional analysis of NHANES 2007–2020

**DOI:** 10.1016/j.pmedr.2025.103043

**Published:** 2025-03-25

**Authors:** Yuhang Liu, Ying Xu, Zhaohong Sun, Siyao Gao

**Affiliations:** a School of Physical Education and Sports, Central China Normal University, Wuhan 430079, PR China; b Department of Hematology, The First Affiliated Hospital, and College of Clinical Medicine of Henan University of Science and Technology, Luoyang 471003, PR China; c College of Physical Education, Chongqing University, Chongqing 401331, PR China; d Department of Physical Education, Central South University, Changsha 410083, PR China

**Keywords:** Physical activity, Sugar-sweetened beverages, Obesity, Young adults, NHANES

## Abstract

**Objective:**

This study aimed to investigate the independent and joint association of sugar-sweetened beverages (SSBs) and physical activity with obesity among young U.S. adults.

**Methods:**

We selected 11,318 U.S. young adults aged 20–44 years from the National Health and Nutrition Examination Survey (2007–2020). Physical activity was self-reported using the Global Physical Activity Questionnaire, while SSBs consumption was calculated from a single day of twenty-four-hour dietary recall. Multivariable logistic regression models, and sensitivity analyses were used to estimate the associations between SSBs, physical activity, and obesity.

**Results:**

There were 4216 cases of obesity (35.5 %). A positive relationship between the consumption of SSBs and the prevalence of obesity was observed, while physical activity was negatively associated with the prevalence of obesity. Relative to the moderate SSBs consumption + inactive participants, those who were insufficiently active [adjusted odds ratio (AOR) = 0.75, 95 % CI: 0.58, 0.97] and physically active (AOR = 0.72, 95 % CI: 0.62, 0.85) had a lower likelihood of obesity among moderate SSBs consumers (1–499 kcal/day). However, this pattern was not found in the heavy SSBs consumers (≥ 500 kcal/day) (*P* > 0.05).

**Conclusions:**

In conclusion, physical activity was associated with a lower prevalence of obesity among moderate SSBs consumers, while this pattern did not observe in heavy SSBs consumers. Further studies are needed to validate these results and determine causality.

## Highlights

• Physical activity was inversely associated with obesity in moderate consumers.
• This association was not observed in heavy consumers.
• Physically active individuals need to limit intake of sugar-sweetened beverages.

## 1 Introduction

Obesity is one of the most serious but preventable public health problems around the world, which stems from the imbalance between energy intake, energy expenditure, and energy turnover (Flier, 2004; Finucane et al., 2011; Boutari and Mantzoros, 2022). Despite current public health efforts, the prevalence of obesity remains stubbornly high (GBD, 2015 *Obesity and Overweight. World health Organization*, 2021). In 2022, overweight and obesity affected 59 % of the adult (43 % overweight, 16 % obesity) aged ≥18 years (World Health Organization, 2022). A large body of evidence indicates that obesity is closely associated with increased all-cause mortality and higher risk of an extensive range of chronic diseases [cardiovascular diseases (CVDs), diabetes mellitus, cancers, and mental disorders] which affect subjects' quality of life and raise the burden of healthcare costs (Anstey et al., 2011; Emerging Risk Factors Collaboration et al., 2011; Flegal et al., 2013).

Notably, the obesity epidemic has been concurrent with excessive consumption of sugar-sweetened beverages (SSBs) across the globe (Malik et al., 2010; Hu and Malik, 2010; Rosinger et al., 2017; Jiang et al., 2022). The SSBs is acknowledged as the largest single source of dietary caloric intake. Generally, SSBs with considerable caloric sweeteners include soft drinks, fruit drinks (not 100 %), energy drinks, and nutritional beverages. Malik et al. underlined that excessive intake of sugary drinks can increase the risk of obesity (Malik et al., 2010). Among more than 4000 participants in the Framingham Offspring Study, those who consumed ≥1 soft drink per day had a 37 % higher risk of obesity compared with infrequent soft drink drinkers (Dhingra et al., 2007). Despite a decline in SSBs intake over the past decade, consumption remains high (Nguyen et al., 2023). For these reasons, the consumption of SSBs is a potentially prominent contributor to obesity around the world. Moreover, the significant benefits of physical activity on health have been well established, and a review revealed the importance of physical activity for the deleterious health effects of fructose (Piercy et al., 2018; Tappy and Rosset, 2019). Thus, the potential role of physical activity in the association between SSBs intake and obesity warrants further analysis.

To our knowledge, there exist limitations in prior related studies as follows: firstly, a systematic review indicated that, after adjustment for energy balance factors, the results of the association between SSBs and risk of obesity remained inconsistent (Trumbo and Rivers, 2014); secondly, the independent association of SSBs, physical activity, and obesity has been extensively studied (Lahti-Koski et al., 2002; Te Morenga et al., 2012). However, the joint association of SSBs and physical activity with the risk of obesity is not fully understood. Whether physical activity could mitigate the detrimental impact of SSBs on obesity remains unclear; thirdly, the majority of prior studies focused on the joint association of SSBs and physical activity on obesity in children and adolescents, while relatively neglected young adult populations. Compared to children, young adults have a higher capacity and potential for consumption, and studies reported that young adults also consume excessive amounts of SSBs (Alafif et al., 2021; Storey et al., 2006); lastly, some methodological limitations require attention. One is that prior research failed to fully account for important confounding variables, such as sedentary behavior, sleep duration, and total energy intake (kcal/day), influencing the individuals' energy balance (Yu et al., 2023). This restricts their findings' comparability and extrapolation. Another limitation is that most of the prior studies had small sample sizes, limiting the generalizability of the findings to the general population (Nikpartow et al., 2012). To compensate for the above limitations, this cross-sectional study utilizing data from the National Health and Nutrition Examination Survey (NHANES) aimed to investigate the independent and joint associations between SSBs consumption, physical activity, and obesity in young U.S. adults.

## 2 Methods

### 2.1 Study population

NHANES is a continuous nationwide cross-sectional study conducted by the Centers for Disease Control and Prevention every two years since 1999 of the U.S. non-institutionalized civilian resident population. The NHANES employed a stratified, multistage probability sampling method to obtain a representative sample, and basic demographic, socioeconomic information, health-related and nutritional status of participants through personal interviews, standardized questionnaires, and physical examinations. The protocol for NHANES was approved by the Ethics Review Board of the National Center for Health Statistics, and all participants provided written informed consent prior to participation. NHANES data are publicly available at http://www.cdc.gov/nchs/nhanes.htm.

**Fig. 1:**
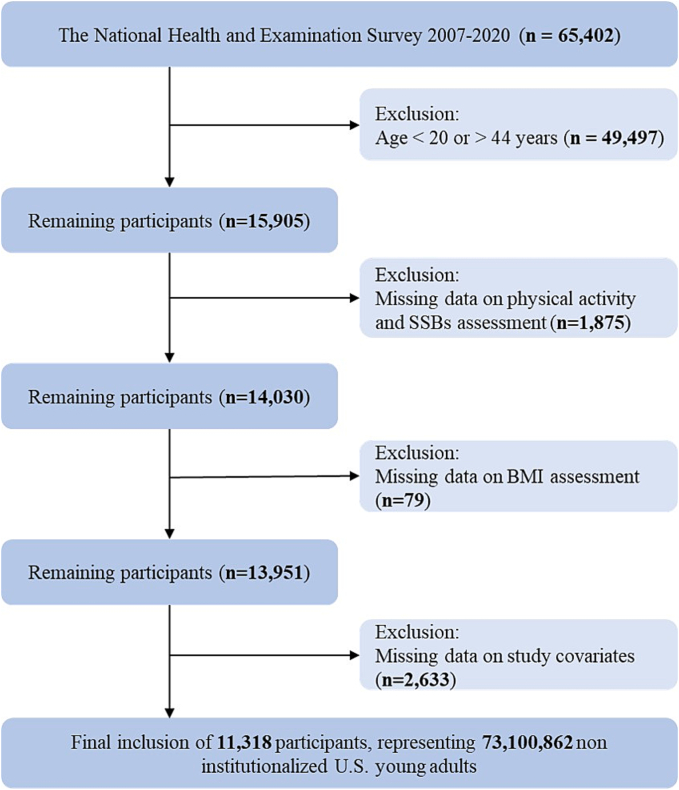
Flowchart of participant selection among young U.S. adults from NHANES 2007–2020. Abbreviations: BMI = Body mass index; SSBs = Sugar-sweetened beverages.

The study initially included 65,402 participants from NHANES 2007 to 2020. Firstly, according to the definition of the age range of young adults from previous studies, we excluded individuals who were younger than 20 years or older than 44 years (*n* = 49,497) (Han and Powell, 2013; Parcha et al., 2022; Shetty et al., 2022). Participants with missing data on the measurement of physical activity, SSBs, and body mass index (BMI) were also excluded (*n* = 1954). We further excluded participants who lacked the data of necessary covariates (*n* = 2633), including poverty index ratio (PIR), sedentary behavior time, sleep duration, energy intake, diabetes mellitus, and on so. Finally, a total of 11,318 NHANES subjects were included in the presented study, representing a weighted 73,100,862 non-institutionalized U.S. population. The process of inclusion and exclusion are shown in Fig. 1.

### 2.2 Measurement of physical activity and SSBs

Physical activity data was obtained from the Global physical activity questionnaire in NHANES (Tian et al., 2022; Liang et al., 2023). Under the Physical Activity Guidelines for Americans, physical activity was categorized into three distinct groups: inactive [engaging in no moderate to vigorous physical activity (MVPA)], insufficiently active [engaging in moderate-intensity physical activity (MPA) < 150 min per week or vigorous-intensity physical activity (VPA) < 75 min per week] and physically active (engaging in MPA ≥ 150 min per week or VPA ≥ 75 min per week).

In this study, the definition of SSBs included the artificially sweetened beverages. Specifically, soft drinks, fruit drinks (not 100 %), sport and energy drinks, nutritional beverages, smoothies, grain drinks, bottled water, carbonated water, and sweetened coffee and tea were defined as SSBs (Zhang et al., 2021). The nutritional information for each food item and its components in the NHANES were computed and converted into United States Department of Agriculture (USDA) codes by the research staff. Sugary drinks with added sugar consumption were identified using the recommended food codes from the USDA Food and Nutrient Database for Dietary Studies (Mesirow and Welsh, 2015; Lin et al., 2020). A prior study established that heavy SSBs consumption was defined as ≥ 500 kcal/day (Xie et al., 2022). Thus, participants were categorized into three groups according to the daily calorie intake from SSBs: non-SSB consumption: participants reported 0 kcal/day from SSBs; moderate SSBs consumption: participants consumed 1–499 kcal/day from SSBs; heavy SSBs consumption: participants consumed 500 kcal/day from SSBs. Moreover, based on the definition from most U.S. studies, a standard 12-oz serving of beverage was defined.

We further created nine categories according to physical activity patterns and SSBs: non-SSB consumption and physically active group; non-SSB consumption and insufficiently active group; non-SSB consumption and inactive group; moderate SSB consumption and physically active group; moderate SSB consumption and insufficiently active group; moderate SSB consumption and inactive group; heavy SSB consumption and physically active group; heavy SSB consumption and insufficiently active group; heavy SSB consumption and inactive group.

### 2.3 Assessment of obesity

At the mobile examination centers, trained health technicians follow standardized procedures outlined in a procedural manual to measure weight, height, waist circumference, and body fat. Each measurement session is carried out by a technician in collaboration with an accompanying recorder. BMI is a widely accepted indicator of obesity status, calculated using the formula: weight (kg) divided by the square of height (m). From a public health perspective, a simple and feasible parameter would make the results of epidemiological studies more easily interpretable and translatable into practice. Thus, according to the definitions of the World Health Organization, participants with a BMI ≥ 30 were classified as having obesity in this study. We also utilized data from the NHANES and selected three distinct anthropometric indices as obesity indicators to employ the sensitivity analyses: waist circumference, body fat percentage (BFP), and weight-adjusted waist index (WWI). Whole body fat was evaluated via X-ray absorptiometry scans conducted on a Hologic Discovery A system, using the APEX 3.2 software. The BFP, encompassing the head, limbs, and trunk areas, was derived to assess the magnitude and distribution of body fat. WWI is calculated as the waist circumference in centimeters divided by the square root of weight (kg).

### 2.4 Definition of covariates

All covariates included demographic, socioeconomic, lifestyle, and health variables. Demographic and socioeconomic variables were collected: Race/ethnicity was categorized as non-Hispanic White, non-Hispanic Black, Mexican American, and other races (including multiracial and other Hispanic). There are three categories of education levels: higher than high school, high school, and less than high school. PIR was defined by Department of Health and Human Services guidelines (< 1.3, 1.3–3.5, and > 3.5). According to their marital status, three groups were classified as widowed/ divorced/separated, never married, or married/living with their partner. Lifestyle and health-related variables were collected: smoking status (never, former, current), drinking status (never, former, current), self-reported sleep duration (hour/day), self-reported sedentary behavior time (min/day), and total energy intake (kcal/day). The diabetes mellitus was defined according to the following criteria: fasting plasma glucose of ≥126 mg/dL, 2-h plasma glucose of ≥200 mg/dL, hemoglobin A1c level of ≥6.5 %, or a self-reported diagnosis of diabetes diagnosed by a professional doctor. Hypertension was defined as an average of three consecutive measurements of systolic blood pressure of ≥140 mmHg and diastolic blood pressure of ≥90 mmHg. CVDs were defined as the presence of a self-reported history, as confirmed by a health professional before the survey, of coronary heart disease, congestive heart failure, heart attack, or stroke. Depression was assessed using the Patient Health Questionnaire, with scores of 10 or above considering the presence of depression.

### 2.5 Statistical analysis

Continuous variables were presented as weighted mean ± standard error (SE), whereas categorical variables were expressed as counts (weighted percentages, %). Chi-square tests were used for categorical variables, and the one-way ANOVA analysis was used for continuous variables. Multivariable logistic regression models were used to estimate adjusted odds ratios (OR) and 95 % confidence intervals (CI) for the independent and joint associations between SSBs, physical activity, and obesity. We used two models: the crude model was not adjusted for any variables. The adjusted model was adjusted age, gender, race/ethnicity, marital status, PIR, education level, drinking status, smoking status, sedentary behavior time (min/day), sleep duration (hour/day), SSBs/physical activity, and energy intake (kcal). Additionally, the interaction analyses were performed to determine whether there was a significant difference. Sensitivity analyses were conducted to validate the robustness of our findings. First, additional covariates of the medical history of diabetes mellitus (yes or no), hypertension (yes or no), CVDs (yes or no), depression (yes or no), and Healthy Eating Index (HEI)-2015 score were added to multivariable logistic regression models to minimize the impact of their possible influences on the outcome. Second, we excluded special diet consumers (such as low-calorie, low-fat, sugar-free, diabetic, and others), and extremely high total energy consumers (5000 kcal/day) in the study population. Finally, our primary findings were evaluated using multivariate linear regression models, with waist circumference, BFP, and WWI selected as obesity indicators (continuous variables). All statistical analyses were performed using the R statistical programming language (X64 version 4.3.1; R Foundation for Statistical Computing). *P* value <0.05 was considered statistically significant.

## 3 Results

The characteristics of participants at baseline by the SSBs consumption and physical activity are presented in Table 1. There were 4216 cases of obesity (35.5 %), and 7102 cases without (65.5 %). The significant differences in gender, race/ethnicity, education, PIR, BMI, sedentary behavior time, MVPA, total energy intake, smoke status, alcohol consumption, hypertension, and CVDs among participants with different statuses of SSBs consumption and physical activity (*P* < 0.01).

**Table 1:** Descriptive characteristics of young U.S. adults aged 20 to 44 years from NHANES 2007–2020 (*n* = 11,318).

Variables	Total	SSBs consumption				Physical activity patterns			
		Non SSBs consumption (0 kcal/day)	Moderate SSBs consumption (1–499 kcal/day)	Heavy SSBs consumption (≥ 500 kcal/day)	*P*-value	Inactive (0 min/week MVPA)	Insufficiently active (< 150 min/week MVPA)	Physically active (≥ 150 min/week MVPA)	*P*-value
No. of participants	11,318 (100.0)	4018 (37.1)	6444 (55.9)	856 (6.9)	–	1875 (14.0)	1248 (10.8)	8195 (75.2)	–
Age	31.7 ± 0.2	31.8 ± 0.2	31.6 ± 0.2	31.3 ± 0.4	0.6	33.0 ± 0.3	33.0 ± 0.4	31.3 ± 0.2	< 0.001

Gender									
Female	5657 (48.3)	2185 (52.9)	3202 (47.6)	270 (29.8)	< 0.001	1211 (61.1)	777 (60.2)	3669 (44.3)	< 0.001
Male	5661 (51.7)	1833 (47.1)	3242 (52.4)	586 (70.2)		664 (38.9)	471 (39.8)	4526 (55.7)	

Race/ethnicity									
Non-Hispanic White	4429 (60.7)	1595 (63.0)	2434 (59.1)	400 (61.7)	< 0.001	579 (49.6)	465 (59.2)	3385 (63.0)	< 0.001
Non-Hispanic Black	2398 (12.0)	747 (10.2)	1458 (12.9)	193 (14.1)		451 (15.2)	265 (12.6)	1682 (11.3)	
Mexican American	1834 (11.5)	499 (8.5)	1203 (13.4)	132 (11.6)		369 (15.7)	190 (10.9)	1275 (10.7)	
Other races	2657 (15.8)	1177 (18.3)	1349 (14.6)	131 (12.6)		476 (19.6)	328 (17.3)	1853 (14.9)	

Education level									
Less than high school	2009 (13.0)	548 (9.4)	1228 (14.2)	233 (22.5)	< 0.001	453 (19.9)	202 (13.2)	1354 (11.7)	< 0.001
High school	2555 (21.8)	664 (15.7)	1616 (24.3)	275 (34.6)		497 (27.3)	267 (22.1)	1791 (20.7)	
Higher than high school	6754 (65.2)	2806 (74.9)	3600 (61.6)	348 (42.9)		925 (52.8)	779 (64.7)	5050 (67.6)	
PIR	2.8 ± 0.0	3.0 ± 0.1	2.7 ± 0.1	2.2 ± 0.1	< 0.001	2.4 ± 0.1	2.8 ± 0.1	2.8 ± 0.1	< 0.001
<1.3	3961 (26.8)	1177 (21.9)	2358 (28.1)	426 (42.3)	< 0.001	790 (34.8)	415 (25.9)	2756 (25.4)	< 0.001
1.3–3.5	4173 (35.6)	1420 (34.5)	2465 (36.5)	288 (33.7)		690 (38.7)	459 (36.6)	3024 (34.8)	
>3.5	3184 (37.7)	1421 (43.6)	1621 (35.4)	142 (24.1)		395 (26.5)	374 (37.5)	2415 (39.8)	

Marital status									
Married/Living with Partner	6422 (56.6)	2246 (54.6)	3682 (57.9)	494 (56.3)	0.1	1126 (61.2)	737 (61.5)	4559 (55.0)	< 0.001
Never married	3863 (34.9)	1419 (37.1)	2165 (33.4)	279 (34.6)		533 (27.8)	386 (28.5)	2944 (37.1)	
Widowed/Divorced/Separated	1033 (8.6)	353 (8.3)	597 (8.7)	83 (9.0)		216 (11.0)	125 (10.0)	692 (7.9)	
BMI	28.7 ± 0.1	28.0 ± 0.2	29.1 ± 0.1	29.2 ± 0.4	< 0.001	30.48 ± 0.25	29.79 ± 0.29	28.21 ± 0.14	< 0.001
Underweight	212 (1.9)	64 (1.7)	129 (2.0)	19 (2.5)	< 0.001	39 (2.1)	33 (2.5)	140 (1.8)	< 0.001
Normal	3586 (33.1)	1448 (36.4)	1889 (31.2)	249 (30.7)		487 (25.6)	364 (28.1)	2735 (35.2)	
Overweight	3304 (29.5)	1200 (30.1)	1882 (29.5)	222 (26.5)		513 (26.1)	354 (29.9)	2437 (30.1)	
Obesity	4216 (35.5)	1306 (31.8)	2544 (37.3)	366 (40.3)		836 (46.2)	497 (39.5)	2883 (32.9)	
Sedentary behavior time (minutes/per day)	366.1 ± 4.2	382.5 ± 5.7	358.3 ± 4.7	341.6 ± 9.3	< 0.001	415.8 ± 7.0	434.5 ± 9.8	347.1 ± 4.5	< 0.001
Sleep hour	7.1 ± 0.0	7.2 ± 0.0	7.1 ± 0.0	6.9 ± 0.1	< 0.05	7.17 ± 0.05	7.22 ± 0.05	7.12 ± 0.03	0.2
Total MVPA (minutes/per week)	1474.4 ± 33.8	1242.7 ± 33.1	1531.3 ± 43.8	2314.2 ± 138.1	< 0.001	0.0 ± 0.0	82.1 ± 1.5	1673.9 ± 37.3	< 0.001
Inactive (0 min/week)	1875 (14.0)	576 (11.7)	1146 (15.2)	153 (16.0)	< 0.001	–	–	–	–
Insufficiently active (0–150 min/week)	1248 (10.8)	446 (10.2)	732 (11.5)	70 (7.7)		–	–	–	
Physically active (≥150 min/week)	8195 (75.2)	2996 (78.1)	4566 (73.2)	633 (76.4)		–	–	–	
Total energy intake (kcal)	2295.3 ± 12.7	2071.5 ± 20.7	2325.6 ± 18.0	3247.6 ± 49.0	< 0.001	2158.4 ± 30.2	2172.8 ± 33.0	2338.3 ± 16.8	< 0.001
SSBs consumption (kcal)	155.6 ± 4.4	0.0 ± 0.0	180.3 ± 2.4	788.2 ± 14.8	< 0.001	176.2 ± 6.6	141.9 ± 8.3	153.7 ± 4.9	< 0.05
Non SSBs consumption (0 kcal/day)	4018 (37.1)	–	–	–	–	153 (7.9)	70 (5.0)	633 (7.1)	< 0.001
Moderate SSBs consumption (1–499 kcal/day)	6444 (55.9)	–	–	–		1146 (61.0)	732 (59.9)	4566 (54.4)	
Heavy SSBs consumption (≥ 500 kcal/day)	856 (6.9)	–	–	–		576 (31.1)	446 (35.2)	2996 (38.5)	

Drinking status									
Never	1294 (9.3)	494 (9.7)	726 (9.2)	74 (8.1)	< 0.05	305 (14.0)	180 (10.4)	809 (8.3)	< 0.001
Former	837 (7.1)	254 (5.9)	487 (7.5)	96 (10.9)		185 (10.5)	109 (8.9)	543 (6.3)	
Current	9187 (83.5)	3270 (84.4)	5231 (83.3)	686 (81.0)		1385 (75.4)	959 (80.8)	6843 (85.5)	

Smoking status									
Never	6982 (60.1)	2706 (65.8)	3910 (58.6)	366 (42.1)	< 0.001	1204 (62.1)	807 (60.5)	4971 (59.7)	< 0.05
Former	1569 (16.2)	573 (16.8)	880 (15.7)	116 (16.2)		217 (12.9)	156 (15.4)	1196 (16.9)	
Current	2767 (23.7)	739 (17.4)	1654 (25.7)	374 (41.7)		454 (25.1)	285 (24.1)	2028 (23.4)	

Hypertension									
No	9202 (82.6)	3368 (85.2)	5183 (81.4)	651 (78.9)	< 0.001	1507 (79.5)	971 (78.1)	6724 (83.9)	< 0.001
Yes	2116 (17.4)	650 (14.8)	1261 (18.6)	205 (21.1)		368 (20.5)	277 (21.9)	1471 (16.1)	

Diabetes mellitus									
Diabetes mellitus	687 (4.7)	214 (4.0)	410 (5.2)	63 (5.3)	0.1	170 (8.0)	101 (6.4)	416 (3.9)	< 0.001
IFG	374 (3.0)	106 (2.5)	227 (3.3)	41 (4.2)		56 (3.2)	45 (4.1)	273 (2.9)	
IGT	269 (2.3)	85 (2.2)	164 (2.3)	20 (2.8)		70 (4.1)	38 (3.1)	161 (1.8)	
No	9988 (89.9)	3613 (91.3)	5643 (89.3)	732 (87.7)		1579 (84.7)	1064 (86.4)	7345 (91.4)	

CVDs									
No	11,093 (98.2)	3949 (98.7)	6315 (98.2)	829 (96.6)	< 0.05	1811 (96.8)	1219 (98.0)	8063 (98.5)	< 0.05
Yes	225 (1.8)	69 (1.3)	129 (1.8)	27 (3.4)		64 (3.2)	29 (2.0)	132 (1.5)	

Footnotes: Continuous variables are presented as weighted mean ± SE, and categorical variables are presented as n (weighted %). *P*-value was calculated by chi-square tests and one-way ANOVA analysis for categorical and continuous variables, respectively.

Abbreviations: BMI = Body mass index; CVDs = Cardiovascular diseases; IFG = Impaired fasting glycaemia; IGT = Impaired glucose tolerance; MVPA = Moderate-to-vigorous physical activity; PIR = Poverty income ratio; SE = Standard error; SSBs = Sugar-sweetened beverages.

As shown in Table 2 and Table S1, participants who consumed moderate SSBs consumption [(Adjusted odd ratios, (AOR) = 1.23, 95 % CI: 1.06, 1.43)] and heavy SSBs (AOR = 1.50, 95 % CI: 1.21, 1.87) were more likely to having obesity compared with the non-SSBs consumers. Compared with participants who never consumed SSBs servings/d, those who consumed >1 to <2 servings/day (AOR = 1.19, 95 % CI: 1.01, 1.41) and ≥ 2 SSBs servings/day (AOR = 1.55, 95 % CI: 1.30, 1.85) had a 19 % and 55 % higher risk of obesity, respectively. Furthermore, the AOR of obesity was 1.05 (95 % CI, 1.02, 1.07) for per 100 kcal/day increase in SSBs consumption, and that of obesity was 1.09 (95 % CI, 1.06, 1.13) for each SSBs serving/day. Compared with inactive participants, insufficiently active participants (AOR = 0.81, 95 % CI: 0.67, 0.99) and physically active participants (AOR = 0.70, 95 % CI: 0.61, 0.80) were significantly associated with lower risks of obesity, respectively.

**Table 2:** Independent and joint associations between sugar-sweetened beverages, physical activity and obesity among young U.S. adults aged 20 to 44 years from NHANES 2007–2020 (*n* = 11,318).

	Crude model	Adjusted model
	OR (95 % CI)	AOR (95 % CI)
SSBs a		
Non SSBs consumption (0 kcal/day)	1.00	1.00
Moderate SSBs consumption (1–449 kcal/day)	1.27 (1.11, 1.46)	1.23 (1.06, 1.43)
Heavy SSBs consumption (> 500 kcal/day)	1.44 (1.18, 1.77)	1.50 (1.21, 1.87)
*P* for trend	< 0.001	< 0.01
Per 100 kcal/d increase	1.04 (1.02, 1.06)	1.05 (1.02, 1.07)

Physical activity patterns b		
Inactive (0 min/week MVPA)	1.00	1.00
Insufficiently active (< 150 min/week MVPA)	0.76 (0.63, 0.92)	0.81 (0.67, 0.99)
Physically active (≥ 150 min/week MVPA)	0.57 (0.50, 0.64)	0.70 (0.61, 0.80)
*P* for trend	< 0.01	< 0.01

SSBs and physical activity patterns c		
Non SSBs consumption group		
Non SSBs consumption and inactive	1.00	1.00
Non SSBs consumption and insufficiently active	0.83 (0.59, 1.17)	0.88 (0.62, 1.26)
Non SSBs consumption and physically active	0.54 (0.41, 0.71)	0.63 (0.47, 0.85)
*P* for trend	< 0.01	< 0.001

Moderate SSBs consumption group		
Moderate SSBs consumption and inactive	1.00	1.00
Moderate SSBs consumption and insufficiently active	0.711 (0.554, 0.91)	0.75 (0.58, 0.97)
Moderate SSBs consumption and physically active	0.584 (0.505, 0.67)	0.72 (0.62, 0.85)
*P* for trend	< 0.01	< 0.01

Heavy SSBs consumption group		
Heavy SSBs consumption and inactive	1.00	1.00
Heavy SSBs consumption and insufficiently active	1.01 (0.48, 2.11)	1.11 (0.54, 2.32)
Heavy SSBs consumption and physically active	0.76 (0.48, 1.21)	0.92 (0.55, 1.52)
*P* for trend	0.20	0.69

Abbreviations: AOR = Adjusted odds ratio; OR = Odds ratio; PIR = Poverty income ratio; SSBs = Sugar-sweetened beverages.

a Adjusted for age, gender, race or ethnicity, marital status, PIR, education level, drinking status, smoking status, sedentary behavior time, sleep duration, physical activity, and energy intake (kcal).

b Adjusted for age, gender, race or ethnicity, marital status, PIR, education level, drinking status, smoking status, sedentary behavior time, sleep duration, SSBs, and energy intake (kcal).

c Adjusted for age, gender, race or ethnicity, marital status, PIR, education level, drinking status, smoking status, sedentary behavior time, sleep duration, and energy intake (kcal).

Relative to the non-SSBs consumption and inactive group, participants who reported non-SSBs consumption and being physically active were significantly associated with the lower odds of obesity (AOR = 0.63, 95 % CI: 0.47, 0.85). Among moderate SSBs consumers, insufficiently active (AOR = 0.75, 95 % CI: 0.58, 0.97) and physically active (AOR = 0.72, 95 % CI: 0.62, 0.85) individuals exhibited a lower likelihood of obesity compared to inactive individuals. However, this relationship between physical activity and obesity was not found in the heavy SSBs consumers (*P* > 0.05). When the non-SSBs consumption and inactive group as a single reference group across nine groups, the associations between “moderate SSBs consumption + insufficiently active”, “moderate SSBs consumption + physically active” and obesity was not significant (*P* > 0.05) after controlling covariates (Table 3).

**Table 3:** Joint associations between sugar-sweetened beverages, physical activity and obesity among young U.S. adults aged 20 to 44 years from NHANES 2007–2020 (n = 11,318).

	Crude model	Adjusted model
	OR (95 % CI)	AOR (95 % CI)
SSBs and physical activity		
Non SSBs consumption + inactive	1.00	1.00
Non SSBs consumption + insufficiently active	0.83 (0.59, 1.17)	0.89 (0.62, 1.27)
Non SSBs consumption + physically active	0.54 (0.41, 0.71)	0.67 (0.50, 0.89)
Moderate SSBs consumption + inactive	1.20 (0.91, 1.57)	1.21 (0.90, 1.63)
Moderate SSBs consumption + insufficiently active	0.85 (0.61, 1.18)	0.91 (0.64, 1.29)
Moderate SSBs consumption + physically active	0.70 (0.54, 0.90)	0.85 (0.64, 1.13)
Heavy SSBs consumption + inactive	1.08 (0.69, 1.69)	1.26 (0.79, 2.02)
Heavy SSBs consumption + insufficiently active	1.10 (0.55, 2.18)	1.29 (0.63, 2.64)
Heavy SSBs consumption + physically active	0.83 (0.60, 1.13)	1.06 (0.75, 1.49)
*P* for trend	0.15	< 0.05

Adjusted model was adjusted for age, gender, race or ethnicity, marital status, PIR, education level, drinking status, smoking status, sedentary behavior time, sleep duration, and energy intake (kcal).

Abbreviations: AOR = Adjusted odds ratio; OR = Odds ratio; PIR = Poverty income ratio; SSBs = Sugar-sweetened beverages.

The results of sensitivity analyses were consistent with our primary analyses. As presented in Table S2, after additionally adjusting for potential history of chronic diseases, HEI-2015 scores, depressive symptoms, and excluding special diet consumers (*n* = 1128) and extremely high total energy consumers (5000 kcal/day) (*n* = 232), our main results remained stable. Furthermore, when we used the other three obesity indicators, including waist circumference, BFP, and WWI as the outcome, respectively, the results did not change substantially (Table S3).

## 4 Discussion

### 4.1 Principal findings

To the best of our knowledge, this is the first study to investigate the joint associations between SSBs consumption, physical activity and obesity in a nationally representative sample of young U.S. adults. The key findings of this study can be summarized as follows: firstly, our analysis revealed a positive association between SSBs consumption and the prevalence of obesity, while physical activity was negatively associated with the prevalence of obesity; secondly, among moderate SSBs consumers, those who were insufficiently active or physically active showed lower odds of being obese, compared to the reference group (moderate SSBs consumption + inactive); thirdly, the inverse association of physical activity with obesity was not observed among heavy SSBs consumers. It should be pointed out that this study adopted a cross-sectional design, which means we can only observe the relationships between SSBs, physical activity, and obesity, but not causations.

### 4.2 Comparison with previous works

In this study, our results supported that there are direct associations between SSBs, physical activity and obesity, in agreement with findings from previous similar studies. Regarding SSBs consumption and obesity, the recent meta-analysis (twenty-one cohorts with 448,661 adults; sixteen randomized controlled trials with 1343 adults) found that each serving/day increase in SSBs consumption was related to a 0.42 kg (95 % CI: 0.26 kg to 0.58 kg) higher body weight in adults (Nguyen et al., 2023). Another meta-analysis in this field to explore dose-response relations between SSBs and obesity (including thirty-nine studies) showed each 250 mL/day increase in consumption of SSBs and artificially sweetened beverages, the risk of obesity increased by 12 % [relative risks (RR) = 1.12, 95 % CI: 1.05–1.19] and 21 % (RR = 1.21, 95 % CI: 1.09–1.35), respectively (Qin et al., 2020). Concerning physical activity, its negative association with obesity has been well documented (Cleven et al., 2020). For instance, a systematic review identified 40 studies concluded that the prevention of weight gain is most pronounced when MVPA is above 150 min/week and the strong relationship between greater time spent in physical activity and attenuated weight gain was also observed (Jakicic et al., 2019).

The findings of joint associations between SSBs, physical activity and the risk of obesity among young U.S. adults are novel. We found that U.S. young adults who reported moderate intake of SSBs and being physically active were less likely to have obesity, relative to the reference group (moderate SSBs consumption + physically inactive). However, this inverse relationship was not observed among heavy SSBs consumers. Our findings are consistent with those of previous similar studies. A population-based NHANES study reported that participants in the highest quartile of servings SSBs had a higher BMI (*P* < 0.05) and a relatively lower proportion of them were physically active, compared to those with other quartiles of SSBs intake (Bermudez and Gao, 2010). Additionally, in students' population, Yu et al. found that participants who reported “SSB consumption and inadequate MVPA” had a higher risk of being overweight/obese (OR = 1.13, 95 % CI = 1.08, 1.18) compared with those with “no SSB consumption and adequate MVPA” (Yu et al., 2023). Of note, a recent longitudinal study based on two U.S. cohorts [the Nurses' Health Study (*N* = 65,730) and the Health Professionals' Follow-up Study (*N* = 39,418)] indicated a higher number of consumed SSBs per day was associated with risk of CVDs regardless of physical activity levels among adults. However, the generalizability of these findings may be constrained due to the majority of participants who were non-Hispanic White health professionals from this study. Our cross-sectional study included other populations and, consistent with the findings of Pacheco et al., corroborated the current recommendations to limit the intake of SSBs even for physically active individuals (Pacheco et al., 2024).

The mechanisms of the role of SSBs in developing obesity are undetermined (Sigala and Stanhope, 2021). Traditionally, SSBs may contribute to weight gain due to low satiety and incomplete compensation of energy at subsequent meals after liquid calorie consumption (DellaValle et al., 2005). A study has shown that weight gain is greater after consuming isocaloric beverages than after consuming solid foods (DiMeglio and Mattes, 2000). Meanwhile, excessive intake of fructose can lead to hepatic de novo lipogenesis, producing very low-density lipoproteins and postprandial triglycerides (Malik and Hu, 2019). Notably, the harmful effects of excess fructose may be further exacerbated by low levels of physical activity, which may explain our results (Trumbo and Rivers, 2014). Another potential mechanism could be that SSBs intake probably promotes hedonic eating through activation of the brain's reward pathways. Evidence suggests that sweetness is linked to heightened responses in the brain's reward regions (Trumbo and Rivers, 2014). Several studies show that brain reward responses to food cues are positively associated with BMI, and abdominal obesity (Luo et al., 2013; Pursey et al., 2014). However, individuals with higher habitual physical activity showed less food reward-related brain activation(Drummen et al., 2019). These findings largely help to explain our results.

### 4.3 Strengths and limitations

The presented study primarily has four strengths. First, this study comprised 11,318 participants from seven cycles of NHANES, representing 73,100,862 non-institutionalized U.S. young adults, which allowed our findings to be generalizable at the population level. Second, we carefully grouped participants based on SSBs consumption and MVPA (min/week). Third, we considered a moderate number of potential confounders, including sedentary behavior time, sleep duration, and total energy intake, which made our results more reliable to some extent. Lastly, several sensitivity analyses were conducted to validate the robustness of the results.

Our study has several limitations that warrant attention. First, due to the nature of the cross-sectional design of this study, we cannot infer the causal relationships and were unable to assess changes in these relationships over time. Thus, our findings should be taken with caution. Second, our dietary data were collected through a single self-reported twenty-four-hour dietary recall interview, and the physical activity data were also self-reported, which is inevitably subject to some degree of recall and self-report bias. In addition, due to the limitations of reported data, participants in this current study may have been misclassified to some extent. Third, we classified into nine groups based on SSBs consumption and physical activity patterns. The unbalanced number of samples between groups after grouping may lead to a larger 95 % CI for the main analyses. Finally, despite our best efforts to select potential confounders, our analysis may still be affected by unmeasured or insufficiently measured confounders (e.g., genetic and environmental factors) (González-Muniesa et al., 2017).

## 5 Conclusions

Our study showed that the SSBs consumption was positively associated with the prevalence of obesity, while physical activity was inversely correlated with lower odds of obesity. Moreover, physical activity was significantly associated with a lower prevalence of obesity among moderate SSBs consumers, while this pattern was not observed in heavy SSBs consumers. Future studies are needed to validate these findings.

## CRediT authorship contribution statement

**Yuhang Liu:** Writing – original draft, Software, Methodology, Formal analysis, Data curation, Conceptualization. **Ying Xu:** Writing – original draft, Methodology, Formal analysis, Data curation. **Zhaohong Sun:** Writing – original draft, Methodology, Formal analysis, Data curation. **Siyao Gao:** Writing – review & editing, Supervision, Project administration, Methodology, Funding acquisition, Formal analysis, Conceptualization.

## Consent for publication

Not applicable.

## Ethics approval and consent to participate

The NHANES protocols were approved by the National Center for Health Statistics Ethics Review Board (Protocol# 2005–06, Protocol #2011–17, and Protocol #2018–01) and all participants provided written informed consent. All methods were carried out in accordance with relevant guidelines and regulations.

## Funding

The work was supported by the 10.13039/501100012325National Social Science Fund of China (No. 24CTY030).

## Declaration of competing interest

The authors declare that they have no known competing financial interests or personal relationships that could have appeared to influence the work reported in this paper.

## Data Availability

The datasets generated and analyzed during the present study are available from the NHANES databases (Available from https://www. cdc.gov/nchs/nhanes/participant.htm).

## Appendix A Supplementary data

Supplementary materials Image 1
