# Supplementary material for: Joint association of physical activity and sugar-sweetened beverages with obesity in young U.S. adults: A cross-sectional analysis of NHANES 2007–2020
Source: Prev Med Rep. 2025 Mar 25;53:103043. doi: 10.1016/j.pmedr.2025.103043 (PMC11999209; doi:10.1016/j.pmedr.2025.103043)
Supplement: Supplementary file 1 — Supplementary materials [file mmc1.docx]

**Supplementary materials**

**Joint association of physical activity and sugar-sweetened beverages with obesity in young U.S. adults: A cross-sectional analysis of NHANES 2007-2020**

Yuhang Liu ^a^, Ying Xu ^b^, Zhaohong Sun ^c^, Siyao Gao ^d*^

* Correspondence: [gaosiyao@csu.edu.cn](mailto:gaosiyao@csu.edu.cn)

^a^ School of Physical Education and Sports, Central China Normal University, Wuhan 430079, P. R. China.

^b^ Department of Hematology, The First Affiliated Hospital, and College of Clinical Medicine of Henan University of Science and Technology, Luoyang, 471003, P. R. China.

^c^ College of Physical Education, Chongqing University, Chongqing, 401331, P. R. China.

^d^ Department of Physical Education, Central South University, Changsha, 410083, P. R. China.

| **Content** | |
| --- | --- |
| **Supplemental figures and tables** | **Page** |
| **Table S1** Associations between daily intakes of sugar-sweetened beverages and obesity among young U.S. adults aged 20 to 44 years from NHANES 2007–2020 (n = 11,318). | 2 |
| **Table S2** Sensitivity analyses for independent and joint association between sugar-sweetened beverages, physical activity and obesity among young U.S. adults aged 20 to 44 years from NHANES 2007–2020. | 3 |
| **Table S3** Independent and joint association between sugar-sweetened beverages, physical activity and obesity indicators among young U.S. adults aged 20 to 44 years from NHANES 2007–2020 (n = 11,318). | 4 |

| **Table S1** Associations between daily intakes of sugar-sweetened beverages and obesity among young U.S. adults aged 20 to 44 years from NHANES 2007–2020 (n = 11,318). | | | |
| --- | --- | --- | --- |
| Daily intakes | Crude model |  | Adjusted model |
|  | OR (95% CI) |  | AOR (95% CI) |
| 0 | 1.00 |  | 1.00 |
| > 0 to < 1 | 1.02 (0.85, 1.23) |  | 0.96 (0.80, 1.16) |
| > 1 to < 2 | 1.23 (1.06, 1.44) |  | 1.19 (1.01, 1.41) |
| ≥ 2 | 1.51 (1.29, 1.76) |  | 1.55 (1.30 ,1.85) |
| *P* for trend | < 0.01 |  | < 0.01 |
| Each serving/d increase | 1.08 (1.05, 1.11) |  | 1.09 (1.06, 1.13) |
| Adjusted model was adjusted for age, gender, race or ethnicity, marital status, PIR, education level, drinking status, smoking status, sedentary behavior time, sleep duration, physical activity, and energy intake (kcal).  Abbreviations: AOR = Adjusted odds ratio; OR= Odds ratio; PIR = Poverty income ratio. | | | |

| **Table S2** Sensitivity analyses for independent and joint association between sugar-sweetened beverages, physical activity and obesity among young U.S. adults aged 20 to 44 years from NHANES 2007–2020. | | | | | | | | | | | | | | | | | | | | |
| --- | --- | --- | --- | --- | --- | --- | --- | --- | --- | --- | --- | --- | --- | --- | --- | --- | --- | --- | --- | --- |
|  | Adjusted HEI-2015 score | |  | Adjusted hypertension | |  | Adjusted diabetes mellitus | |  | Adjusted CVDs | |  | Adjusted depression | |  | Excluded 1,128 special diet consumers ^d^ | |  | Excluded 232 extremely high total energy consumers | |
|  | AOR (95% CI) | |  | AOR (95% CI) | |  | AOR (95% CI) | |  | AOR (95% CI) | |  | AOR (95% CI) | |  | AOR (95% CI) | |  | AOR (95% CI) | |
| SSBs ^a^ |  |  |  |  |  |  |  |  |  |  |  |  |  |  |  |  |  |  |  |  |
| Non SSBs consumption | 1.00 | |  | 1.00 | |  | 1.00 | |  | 1.00 | |  | 1.00 | |  | 1.00 | |  | 1.00 | |
| Moderate SSBs consumption | 1.10 (0.94, 1.29) | |  | 1.20 (1.04, 1.39) | |  | 1.21 (1.05, 1.41) | |  | 1.23 (1.06, 1.43) | |  | 1.24 (1.07, 1.43) | |  | 1.23 (1.06, 1.42) | |  | 1.24 (1.07, 1.43) | |
| Heavy SSBs consumption | 1.27 (1.00, 1.63) | |  | 1.47 (1.19, 1.81) | |  | 1.47 (1.17, 1.84) | |  | 1.50 (1.20, 1.86) | |  | 1.49 (1.20, 1.85) | |  | 1.52 (1.21, 1.90) | |  | 1.44 (1.15, 1.79) | |
| Physical activity patterns ^b^ |  |  |  |  |  |  |  |  |  |  |  |  |  |  |  |  |  |  |  |  |
| Inactive | 1.00 | |  | 1.00 | |  | 1.00 | |  | 1.00 | |  | 1.00 | |  | 1.00 | |  | 1.00 | |
| Insufficiently active | 0.78 (0.63, 0.97) | |  | 0.79 (0.65, 0.96) | |  | 0.81 (0.66, 0.99) | |  | 0.82 (0.67, 0.99) | |  | 0.82 (0.67, 1.00) | |  | 0.74 (0.60, 0.91) | |  | 0.82 (0.67, 1.00) | |
| Physically active | 0.73 (0.63, 0.85) | |  | 0.71 (0.63, 0.81) | |  | 0.73 (0.63, 0.83) | |  | 0.70 (0.62, 0.80) | |  | 0.71 (0.62, 0.81) | |  | 0.67 (0.59, 0.78) | |  | 0.70 (0.61, 0.80) | |
| SSBs and physical activity patterns ^c^ |  |  |  |  |  |  |  |  |  |  |  |  |  |  |  |  |  |  |  |  |
| Non SSBs consumption group |  |  |  |  |  |  |  |  |  |  |  |  |  |  |  |  |  |  |  |  |
| Non SSBs consumption and inactive | 1.00 | |  | 1.00 | |  | 1.00 | |  | 1.00 | |  | 1.00 | |  | 1.00 | |  | 1.00 | |
| Non SSBs consumption and insufficiently active | 0.83 (0.55, 1.25) | |  | 0.89 (0.61, 1.31) | |  | 0.90 (0.62, 1.32) | |  | 0.88 (0.62, 1.26) | |  | 0.94 (0.66, 1.33) | |  | 0.84 (0.59, 1.19) | |  | 0.88 (0.61, 1.26) | |
| Non SSBs consumption and physically active | 0.67 (0.47, 0.95) | |  | 0.64 (0.47, 0.88) | |  | 0.64 (0.47, 0.88) | |  | 0.63 (0.47, 0.85) | |  | 0.66 (0.49, 0.89) | |  | 0.61 (0.44, 0.84) | |  | 0.63 (0.47, 0.85) | |
| Moderate SSBs consumption group |  |  |  |  |  |  |  |  |  |  |  |  |  |  |  |  |  |  |  |  |
| Moderate SSBs consumption and inactive | 1.00 | |  | 1.00 | |  | 1.00 | |  | 1.00 | |  | 1.00 | |  | 1.00 | |  | 1.00 | |
| Moderate SSBs consumption and insufficiently active | 0.72 (0.55, 0.94) | |  | 0.73 (0.57, 0.93) | |  | 0.73 (0.58, 0.93) | |  | 0.75 (0.58, 0.97) | |  | 0.75 (0.58, 0.97) | |  | 0.67 (0.51, 0.88) | |  | 0.76 (0.59, 0.98) | |
| Moderate SSBs consumption and physically active | 0.75 (0.63, 0.88) | |  | 0.74 (0.63, 0.86) | |  | 0.75 (0.64, 0.87) | |  | 0.72 (0.62, 0.85) | |  | 0.73 (0.62, 0.86) | |  | 0.68 (0.58,0.81) | |  | 0.73 (0.62, 0.85) | |
| Heavy SSBs consumption group |  |  |  |  |  |  |  |  |  |  |  |  |  |  |  |  |  |  |  |  |
| Heavy SSBs consumption and inactive | 1.00 | |  | 1.00 | |  | 1.00 | |  | 1.00 | |  | 1.00 | |  | 1.00 | |  | 1.00 | |
| Heavy SSBs consumption and insufficiently active | 1.26 (0.57, 2.76) | |  | 0.90 (0.39, 2.05) | |  | 1.22 (0.57, 2.62) | |  | 1.17 (0.56, 2.45) | |  | 1.10 (0.52, 2.30) | |  | 0.83 (0.39, 1.78) | |  | 1.17 (0.55, 2.45) | |
| Heavy SSBs consumption and physically active | 0.96 (0.57, 1.62) | |  | 0.94 (0.55, 1.60) | |  | 1.05 (0.63, 1.74) | |  | 0.96 (0.57, 1.62) | |  | 0.93 (0.56, 1.54) | |  | 0.91 (0.53, 1.56) | |  | 0.87 (0.50, 1.49) | |
| ^a^ Adjusted for age, gender, race or ethnicity, marital status, PIR, education level, drinking status, smoking status, sedentary behavior time, sleep duration, physical activity, energy intake (kcal), and HEI-2015 score/hypertension/Diabetes mellitus/CVDs/Depression.  ^b^ Adjusted for age, gender, race or ethnicity, marital status, PIR, education level, drinking status, smoking status, sedentary behavior time, sleep duration, SSBs, energy intake (kcal) and HEI-2015 score/hypertension/Diabetes mellitus/CVDs/Depression. ^c^ Adjusted for age, gender, race or ethnicity, marital status, PIR, education level, drinking status, smoking status, sedentary behavior time, sleep duration, energy intake (kcal), and HEI-2015 score/hypertension/Diabetes mellitus/CVDs/Depression. ^d^ Including low calorie, low fat, sugar free, diabetic, and others. Abbreviations: AOR = Adjusted odds ratio; CVDs = Cardiovascular diseases; HEI-2015 = Healthy eating index-2015 ;OR= Odds ratio; PIR = Poverty income ratio; SSBs=Sugar-sweetened beverages. | | | | | | | | | | | | | | | | | | | | |

| **Table S3** Independent and joint association between sugar-sweetened beverages, physical activity and obesity indicators among young U.S. adults aged 20 to 44 years from NHANES 2007–2020 (n = 11,318). | | | | | |
| --- | --- | --- | --- | --- | --- |
|  | Waist circumference |  | BFP |  | WWI |
|  | β-coefficient (95% CI) |  | β-coefficient (95% CI) |  | β-coefficient (95% CI) |
| SSBs ^a^ |  |  |  |  |  |
| Non SSBs consumption | 1.00 |  | 1.00 |  | 1.00 |
| Moderate SSBs consumption | 2.31 (1.27, 3.34) |  | 0.81 (0.23, 1.38) |  | 0.11 (0.06, 0.15) |
| Heavy SSBs consumption | 3.10 (1.10, 5.11) |  | 0.89 (-0.13, 1.91) |  | 0.14 (0.06, 0.22) |
| *P* for trend | < 0.01 |  | < 0.05 |  |  |
| Physical activity patterns ^b^ |  |  |  |  |  |
| Inactive | 1.00 |  | 1.00 |  | 1.00 |
| Insufficiently active | -1.20 (-2.76, 0.35) |  | -0.57 (-1.43, 0.27) |  | -0.05 (-0.11, 0.01) |
| Physically active | -2.83 (-3.95, -1.71) |  | -2.78 (-3.43, -2.13) |  | -0.22 (-0.27, -0.18) |
| *P* for trend | < 0.01 |  | < 0.01 |  |  |
| SSBs and physical activity patterns ^c^ |  |  |  |  |  |
| Non SSBs consumption group |  |  |  |  |  |
| Non SSBs consumption and inactive | 1.00 |  | 1.00 |  | 1.00 |
| Non SSBs consumption and insufficiently active | 0.44 (-2.30, 3.19) |  | 0.44 (-2.30, 3.19) |  | -0.01 (-0.12, 0.09) |
| Non SSBs consumption and physically active | -3.13 (-5.27, -0.98) |  | -3.13 (-5.27, -0.98) |  | -0.17 (-0.26, -0.07) |
| *P* for trend | < 0.01 |  | < 0.05 |  |  |
| Moderate SSBs consumption group |  |  |  |  |  |
| Moderate SSBs consumption and inactive | 1.00 |  | 1.00 |  | 1.00 |
| Moderate SSBs consumption and insufficiently active | -2.27 (-4.36, -0.17) |  | -1.04 (-2.07, -0.02) |  | -0.06 (-0.13, 0.00) |
| Moderate SSBs consumption and physically active | -3.66 (-5.26, -2.07) |  | -1.31 (-2.08, -0.55) |  | -0.17 (-0.23, -0.12) |
| *P* for trend | < 0.01 |  | < 0.05 |  |  |
| Heavy SSBs consumption group |  |  |  |  |  |
| Heavy SSBs consumption and inactive | 1.00 |  | 1.00 |  | 1.00 |
| Heavy SSBs consumption and insufficiently active | 0.42 (-6.91, 7.76) |  | 0.86 (-2.86, 4.60) |  | -0.03 (-0.33, 0.27) |
| Heavy SSBs consumption and physically active | -1.29 (-6.13, 3.55) |  | 0.55 (-2.13, 3.24) |  | -0.13 (-0.31, 0.04) |
| *P* for trend | 0.54 |  | 0.70 |  | 0.11 |
| ^a^ Adjusted for age, gender, race or ethnicity, marital status, PIR, education level, drinking status, smoking status, sedentary behavior time, sleep duration, physical activity, and energy intake (kcal).  ^b^ Adjusted for age, gender, race or ethnicity, marital status, PIR, education level, drinking status, smoking status, sedentary behavior time, sleep duration, SSBs, and energy intake (kcal).  ^c^ Adjusted for age, gender, race or ethnicity, marital status, PIR, education level, drinking status, smoking status, sedentary behavior time, sleep duration, and energy intake (kcal). Abbreviations: BFP, Body fat percentage; PIR = Poverty income ratio; SSBs=Sugar-sweetened beverages; WWI, Weight-adjusted waist index. | | | | | |
